# The construct validity and responsiveness of the EQ-5D-5L, AQL-5D and a bespoke TTO in acute asthmatics

**DOI:** 10.1007/s11136-019-02345-z

**Published:** 2019-11-02

**Authors:** Christina-Jane Crossman-Barnes, Tracey Sach, Andrew Wilson, Garry Barton

**Affiliations:** Norwich Medical School, University of East Anglia, Norwich, UK

**Keywords:** Asthma, Psychometric, Preference-based, Quality of life, Construct validity, Responsiveness

## Abstract

**Purpose:**

Measuring quality of life in acute asthmatics is challenging, especially when asthma attacks can occur sporadically. Several questionnaires can be used to measure quality of life in this patient group; however, psychometric testing is limited on questionnaires that can be used to estimate Quality Adjusted Life years. The objective of this study is to assess the construct validity (convergent and discriminative validity) and responsiveness of the EuroQol-5-Dimensions 5-Level (EQ-5D-5L), Asthma Quality of Life Utility Index-5 Dimensions (AQL-5D) and Time Trade-Off (TTO) in acute asthma patients.

**Methods:**

Data from a prospective cohort study were used to test the validity and responsiveness of the EQ-5D-5L, AQL-5D and TTO in asthma patients who were recruited from UK accident & emergency departments or hospital wards. The spearman’s rank correlation coefficient, the Kruskal–Wallis test statistic and the standardized response mean were used to test for convergent validity, discriminative validity and responsiveness, respectively.

**Results:**

One hundred and twenty-one participants were included in the available case analysis. The EQ-5D-5L and AQL-5D showed moderate to strong correlations for convergent validity at baseline, week 4 and week 8. The AQL-5D and TTO showed moderate correlations at week 4 and week 8. No statistical significance was observed for discriminative validity at baseline. Both the EQ-5D-5L and the AQL-5D also showed that they were sensitive to change for the recovery responses.

**Conclusions:**

The EQ-5D-5L and AQL-5D showed stronger construct validity and responsiveness compared to the TTO. Therefore, both the EQ-5D-5L and AQL-5D should be considered for use in future economic evaluations.

**Electronic supplementary material:**

The online version of this article (10.1007/s11136-019-02345-z) contains supplementary material, which is available to authorized users.

## Introduction

Patient-reported outcome measures (PROMs) are used widely in research as they are useful for capturing patient’s perceptions for different health states [1]. The PROMs can be disease-specific (particular health-related quality of life aspects relating to people with a specific disease or condition) or generic (particular health-related aspects relating to people in general) questionnaires, and they can be conducted in different modes and at different time points [2].

As seen in a previous systematic review [3], the use of PROMs in asthma is extensive. Many different PROMs have been used for asthma studies [3–6], the Asthma Quality of Life Questionnaire (AQLQ) [7], mini-AQLQ [8], St George’s Respiratory Questionnaire (SGRQ) [9] and EuroQol-5-Dimensions (EQ-5D) [10] being some common ones. As there are so many different PROMs available to use, it is important that there is transparency for their use and knowledge as to which one is more appropriate. However, there is no current guidance on the most appropriate PROMs for asthma [5].

Testing preference-based measures (measures that describe aspects of a patient’s health, which usually consist of several domains, the scores from which are converted into index scores using an algorithm and typically have a scale where 0 is death and 1 is full health) [11] through psychometric techniques have been performed before in many different diseases. However, this is limited in asthma where reliability, validity and responsiveness are often tested on non-preference-based measures [12–18]. A previous study argued that the EQ-5D is valid and reliable for use on asthma patients to help clinicians form better decisions [19]. However, different preference-based measures have not been tested for asthma-related crisis events and previous asthma studies are often based on the older 3-level (3L) version of the EQ-5D, rather than the new 5L version. Therefore, this study aims to test the construct validity and responsiveness of the EQ-5D-5L [20], AQL-5D [21] and Time Trade-Off (TTO) [22] questionnaires. These specific questionnaires were chosen because they are preference-based. They are based on the responses of a sample of the UK public and enable the relative weight of different aspects of quality of life (QoL) to be considered such that QoL can be summarized on a 0 (death) to 1 (full health) utility scale.

## Methods

### Study design and recruitment

This work is based on a prospective observational cohort study (Clinical Trials Number: NCT02771678) that recruited patients who had an asthma-related crisis event (accident and emergency attendance or hospital admission) at one of three sites (Norfolk and Norwich University Hospital, University Hospital Birmingham and Aberdeen Royal Infirmary) [23]. Participants met the inclusion criteria for recruitment if they were adults who had an asthma attack, were not hypoxaemic and did not need help from a carer to complete the questionnaires. One hundred and twenty-one patients were recruited from the hospitals and were asked to complete several quality of life questionnaires over an 8-week period [23]. All participants provided written informed consent, and the study was approved by the NHS Cambridge South Research ethics committee (REC Reference: 16/EE0023). More details on the recruitment, methodology and measures applied in this study are reported elsewhere [23].

### EuroQol-5-Dimensions 5-Level (EQ-5D-5L)

Participants were asked to complete the EQ-5D-5L weekly over the 8-week time period. The EQ-5D-5L has questions about mobility, self-care, usual activities, pain/discomfort and anxiety/depression. Each of these questions has 5 levels to choose from when completing the questionnaire [20]. These levels are no problems, slight problems, moderate problems, severe problems and extreme problems/unable. Once all questions had been answered, then the responses were converted into a health utility score on a 0 (death) to 1 (full health) scale [10]. A visual analogue scale (VAS) is also part of the questionnaire and is a scale which ranges from 0 (the worst health you can imagine) to 100 (the best health you can imagine).

### Asthma Quality of Life Utility Index-5-Dimensions (AQL-5D)

The AQL-5D is a utility index scale generated from the asthma quality of life questionnaire (AQLQ) [7]. Participants were asked to complete the AQLQ monthly over the 8-week period. It has 32 asthma-related questions with a choice of seven different responses for each question. Responses from five questions on the AQLQ were taken to generate the AQL-5D on a utility scale of 0 to 1 [21]. The responses taken were related to questions around sleep, concern, breath, pollution and activity.

### Time Trade-Off (TTO)

The TTO was modified to include two anchor options of ‘current asthma health state’ and ‘well-controlled asthma’, and participants were asked to complete it monthly over the 8-week period [24]. The ‘well-controlled asthma’ health state was described as ‘no asthma symptoms, no limitation of activities due to asthma and no asthma attacks’. This modification was undertaken with a view to identify whether participants had reached their normal well-controlled asthma health state by week 4 or week 8 after having their asthma-related crisis event, and to estimate the utility loss specifically associated with an asthma crisis event excluding the effect of any comorbidities.

The TTO was initially asked during a face-to-face consultation at baseline using a laptop for visual aid. At the week-4 and week-8 follow-ups, the TTO was either asked at a face-to-face consultation during the participant’s routine hospital appointment, or over the telephone, if the follow-ups did not coincide with the participant’s routine hospital appointment. Iterative questioning was used for the TTO, starting at the mid-point of the participant’s estimated remaining life expectancy and increasing or decreasing by 10% increments depending on the response of the participant. For example, participants were asked whether they would choose their ‘current asthma’ health state or ‘well-controlled asthma’ health state at the mid-point value, and if they chose the latter option, their years would decrease by 10% until indifference was achieved. Alternatively, if participants chose the ‘current asthma’ health state option, then their years would increase by 10% until indifference was achieved.

The TTO was chosen as it is the direct valuation method that was used for the EQ-5D, at the time of the study’s conception [25]. The modified TTO is used in this study as it enabled us to consider whether the individual had returned to their well-controlled asthma state, and thereby specifically estimate the loss in utility score associated with an asthma crisis event. In contrast, were a patient to have a utility score below full health according to the EQ-5D, it would be unclear whether they had, e.g. a comorbidity but had fully recovered from the asthma event, or instead were still yet to recover to their well-controlled asthma state. The use of the EQ-5D in the latter situation could lead to the loss in utility score associated with an asthma crisis event being underestimated.

### Construct validity

Construct validity assesses whether the constructs of an instrument are measuring what it should be measuring [26]. Two forms of construct validity were considered: convergent and discriminative validity.

Convergent validity addresses the level of correlation between constructs and instruments. It shows whether the constructs or instruments that are being compared are related to each other as expected. These relations may be strong or weak correlations depending on the relationship expected between the constructs or instruments compared [27].

The correlations for convergent validity have been assessed at baseline, week 4 and week 8 of the study. The Spearman’s rank correlation coefficient was used to determine the correlations with statistical significance considered at the 5% level. Additionally, correlations were considered weak if < 0.3, moderate if 0.3 to 0.5 and strong if > 0.5 [28].

Discriminative validity (also known as known-group validity) is another type of construct validity which has been considered in this analysis [27]. When groups are expected to differ between each other, a test can be conducted to help assess whether a measure can discriminate between the groups. The analysis was conducted based on specific groups that were anticipated to have different levels of utility at baseline due to their different asthma severity. Three peak expiratory flow (PEF) groups were chosen to conduct this analysis, where the PEF was recorded by medical report using a PEF meter [29]:

- < 50% of the best/predicted PEF (life threatening or acute severe asthma)
- 50–75% of the best/predicted PEF (moderate acute asthma)
- > 75% of the best/predicted PEF (good/very good asthma)

The Kruskal–Wallis test statistic was used to conduct the test for discriminative validity across the instruments using the above three PEF subgroups, i.e. to test for a significant difference in mean rank scores between groups. *p*-values were used to display the statistical significance.

### Responsiveness

This was an assessment of all of the quality of life questionnaires, including the PEF to detect any sensitivity to change [26]. The idea behind responsiveness is that it should highlight whether the instruments are measuring the constructs as it should, e.g. by detecting whether an expected improvement or deterioration over a period of time is reflected in the scores for that instrument. Accordingly, we compared groups based on the following question which was asked at week 4 of the study:

- *Question* Compared to your asthma state when you were in hospital approximately 4 weeks ago, how would you rate your asthma now?
- *Answers options* very good, good, moderate, poor or very poor.

Responses to the above question were grouped into 4 categories. As none of the participants chose the last item, ‘*very poor*’, this category was omitted from the groups. Wilcoxon signed-rank tests were conducted to identify any significant changes in scores within each category, accompanied with effect size (ES) and standard response mean (SRM) calculations [27].

$${\text{Effect}}\;{\text{ size }}\;\left( {\text{ES}} \right):\frac{{{\text{Mean}} \;{\text{change}}}}{{{\text{Standard }}\;{\text{deviation}}\; {\text{at}}\; {\text{baseline}}}}$$

$${\text{Standardised }}\;{\text{Response}}\;{\text{ Mean}}\; \, \left( {\text{SRM}} \right):\frac{{{\text{Mean }}\;{\text{change}}}}{{{\text{Standard}} \;{\text{deviation}} \;{\text{of}}\; {\text{change}}}}$$

The SRM helped to indicate how responsive the questionnaires were to change where a higher value would be expected for asthmatics who rated their asthma state as very good compared to 4 weeks ago. Values ranging between 0.20 and 0.50 were considered small, 0.50 to 0.80 were considered moderate and greater than 0.80 were considered large [30].

## Results

The available case analysis was used for this study, where 121 participants were recruited, 42 were lost to follow-up and 8 withdrew [23]. Table 1 shows the baseline participant characteristics of the available case analysis. The participants were recruited from three hospital sites in the UK (Norwich, Birmingham and Aberdeen) in 1 year, which increased the generalizability of the study [23]. The sample size, participant characteristics and attrition rate are similar to other asthma studies which assess the validity of PROMs, which further confirms the representativeness and generalizability of the data [31, 32]. As a sensitivity analysis, a complete case analysis (participants who completed all measures, at all three time points, *N* = 44) was undertaken, where it can be seen they had similar characteristics to the available case sample (see Supplementary Table 1).

**Table 1:** Baseline characteristics of participants for available case analysis

Demographics	*N* = 121
Age (mean, years)	49.68
Height (mean, cm)	167.22
Weight (mean, kg)	85.54
Gender (%)	
Male	26.45
Female	73.55
Ethnicity (%)	
White	95.83
Mixed white and black	0.83
White other	3.33
Smoking status (%)	
Never	42.50
Non smoker	1.67
Smoker	15.00
Ex-smoker	40.83
Highest level of education (%)	
School	47.06
College	33.61
Degree	19.33
Employment status (%)	
Full-time	27.50
Part-time	15.83
Retired	28.33
Stay at home parents	7.50
Student	3.33
Unemployed	17.50

### Convergent validity

The convergent validity for baseline, week 4 and week 8 is shown in Tables 2, 3 and 4, respectively, using Spearman’s rank correlation coefficients for the preference-based measures.

**Table 2:** Convergent validity at baseline using Spearman’s rank correlation coefficient

	EQ-5D-5L (utility)	AQL-5D (utility)	TTO (utility)
EQ-5D-5L (utility)	*N* = 120		
	1.0000^a^		
AQL-5D (utility)	*N* = 118	*N* = 118	
	0.3888^a^**	1.0000^a^	
TTO (utility)	*N* = 111	*N* = 109	*N* = 112
	0.1287^a^	0.0864^a^	1.000^a^

**p value is < 0.01 therefore statistically significant at the 1% level

^a^Pairwise correlation coefficients displayed. Correlation coefficients considered < 0.3 are weak, 0.3 to 0.5 are moderate and > 0.5 are strong

**Table 3:** Convergent validity at week 4 using Spearman’s rank correlation coefficient

	EQ-5D-5L (utility)	AQL-5D (utility)	TTO (utility)	PEF
EQ-5D-5L (utility)	*N* = 71			
	1.0000^a^			
AQL-5D (utility)	*N* = 63	*N* = 70		
	0.5355^a^**	1.0000^a^		
TTO (utility)	*N* = 62	*N* = 62	*N* = 87	
	0.1771^a^	0.3027^a^*	1.000^a^	

*p-value is < 0.05, **p-value is < 0.01 therefore statistically significant at the 5% level and 1% level, respectively

^a^Pairwise correlation coefficients displayed. Correlation coefficients considered < 0.3 are weak, 0.3 to 0.5 are moderate and > 0.5 are strong

**Table 4:** Convergent validity at week 8 using Spearman’s rank correlation coefficient

	EQ-5D-5L (utility)	AQL-5D (utility)	TTO (utility)
EQ-5D-5L (utility)	*N* = 64		
	1.0000^a^		
AQL-5D (utility)	*N* = 61	*N* = 64	
	0.6260^a^**	1.0000^a^	
TTO (utility)	*N* = 60	*N* = 58	*N* = 80
	0.1871^a^	0.3087^a^*	1.000^a^

*p-value is < 0.05, **p-value is < 0.01 therefore statistically significant at the 5% level and 1% level, respectively

^a^Pairwise correlation coefficients displayed. Correlation coefficients considered < 0.3 are weak, 0.3 to 0.5 are moderate and > 0.5 are strong

At baseline, the relationship between the EQ-5D-5L and the AQL-5D were significant at the 1% level, whilst the EQ-5D-5L and the TTO, and the AQL-5D and the TTO were not significantly correlated.

The convergent validity relationships highlighted at baseline had become stronger at week 4. The EQ-5D-5L and the AQL-5D improved to have a strong correlation at the 1% level. The AQL-5D and the TTO had a better relationship at week 4, with a moderate correlation observed at the 5% level.

The same relationships were also observed for the convergent validity at week 8 of the study, as compared to the convergent validity relationships observed at week 4 of the study. Both the EQ-5D-5L and the AQL-5D, and the AQL-5D and the TTO showed the same relationship.

### Discriminative validity

Table 5 shows the discriminative validity test based on three PEF groups. The PEF groups were split into categories of different asthma severities: < 50% of best/predicted PEF, 50–75% of best/predicted PEF and > 75% of best/predicted PEF [29]. The lower proportion (< 50% of best/predicted PEF) indicates that the participants are furthest away from their best or predicated PEF, indicating that they are more poor (have life threatening asthma or acute severe asthma) than the participants who have a PEF of > 75% of their best or predicted PEF (good or very good asthma).

**Table 5:** Discriminative (known-group) validity at baseline using three PEF subgroups (^a^Kruskal–Wallis test conducted and PEF split into three subgroups: < 50% of PEF best/predicted = life threatening/acute severe asthma; 50–75% of PEF best/predicted = moderate acute asthma and > 75% of best/predicted asthma for good/very good asthma [29]) against preference-based measures

	< 50% of best/predicted PEF mean rank^a^	< 50% of best/predicted PEF *N*	50–75% of best/predicted PEF mean rank^a^	50–75% of best/predicted PEF *N*	> 75% of best/predicted PEF mean rank^a^	> 75% of best/predicted PEF *N*	*p*-value*
EQ-5D-5L utility	53.64	18	57.02	42	71.89	27	0.105
AQL-5D utility	53.36	18	56.45	42	68.96	26	0.223
TTO utility	49.91	16	58.11	40	56.70	28	0.713

*No statistical significant difference found for all PROMs between the three PEF subgroups

^a^Mean rank: The utilities are ranked from lowest to highest, and an average is taken based on the ranked order of utilities. The group with the lowest mean rank have the greatest number of lower utility values, and the group with the highest mean rank have the greatest number of higher utility values

Most of the participants were within the 50–75% of best/predicted PEF category indicating that they had moderate acute asthma at baseline. Interestingly, at baseline, some participants were in the third PEF category which indicated they had good/very good asthma based on their PEF being > 75% of their best/predicted value. Even though, most of the mean rank utility values were increasing as hypothesized from the lowest PEF group (< 50% of best/predicted PEF) to the highest PEF group (> 75% of best/predicted PEF), the change was not very large and the results showed no statistical significant differences between any of the preference-based measures displayed.

### Responsiveness

The responsiveness test was conducted and observed how participants felt at week 4 of the study compared to how they were at baseline when in hospital with their asthma-related crisis event. Table 6 shows the results of the changes in means between baseline and week 4. As hypothesized, most of the utilities demonstrated sensitivity to change (which is highlighted from the SRM values). The range for the mean change from poor to very good groups in the EQ-5D-5L utility was from − 0.276 to 0.175, for the AQL-5D from − 0.0065 to 0.169 and for the TTO from − 0.173 to 0.254. The TTO was the only preference-based measure which didn’t have a large sensitivity to change in any of the four groups (poor, moderate, good and very good). Conversely, the AQL-5D showed large responsiveness to change for the very good and good group, and the EQ-5D-5L showed large responsiveness for the good and poor groups.

**Table 6:** Responsiveness of all preference-based measures between baseline and week 4 for recovery rates

Items	*N*	Baseline (mean)	Week 4 (mean)	Mean change	SD at baseline	SD at change	ES	SRM^b^	*p* value^a^
EQ-5D-5L									
Very good	14	0.747	0.922	0.175	0.280	0.235	0.625	0.745**	**0.011**
Good	17	0.585	0.807	0.221	0.325	0.233	0.680	0.948***	**0.000**
Moderate	24	0.630	0.724	0.094	0.265	0.239	0.355	0.393*	**0.031**
Poor	8	0.604	0.328	− 0.276	0.148	0.268	− 1.865	− 1.030***	0.066
AQL-5D									
Very good	15	0.629	0.798	0.169	0.135	0.187	1.252	0.904***	**0.010**
Good	17	0.621	0.787	0.166	0.132	0.140	1.258	1.186***	**0.001**
Moderate	26	0.560	0.621	0.061	0.113	0.110	0.540	0.555**	**0.023**
Poor	9	0.529	0.524	− 0.005	0.107	0.019	− 0.047	− 0.263*	0.356
TTO									
Very good	14	0.679	0.932	0.254	0.250	0.329	1.016	0.772**	**0.014**
Good	19	0.682	0.908	0.227	0.296	0.320	0.767	0.709**	**0.013**
Moderate	23	0.598	0.787	0.189	0.297	0.348	0.636	0.543**	**0.008**
Poor	8	0.881	0.708	− 0.173	0.177	0.376	− 0.977	− 0.471*	0.468

*ES* effect size (mean change/SD at baseline)

*Small change, small responsiveness

**Moderate change, moderately responsive

***Large change, largely responsive

^a^Wilcoxon signed-rank test conducted and *p*-values in bold are statistically significant at the 5% level

^b^*SRM* standardized response mean (mean change/SD of change). If SRM = 0.2 to 0.50 equals small, 0.50 to 0.80 equals moderate and 0.80 and above equals large

## Discussion

This study used psychometric techniques to analyse the construct validity and responsiveness relationships between preference-based measures for people with acute asthma. The data used for this analysis was the available case analysis from the prospective cohort data collected from across three hospital sites in the UK from when people attended A&E or were admitted to hospital with acute asthma symptoms. This study analysed the observed findings by comparing the preference-based measures at three main time points during the study, which were baseline, week 4 and week 8.

### Summary of findings

The correlations between the preference-based measures were mostly moderately to strongly correlated and had strengthened from time points at baseline, through to week 4 and week 8. At baseline, the EQ-5D-5L and the AQL-5D were significantly correlated at the 1% level. As the study progressed, the TTO and AQL-5D also showed more of an association, reaching statistical significance at the 5% level at week 4 and week 8 of the study, suggesting that the TTO and AQL-5D were correlating better when the participants were nearer to their recovery from their asthma-related crisis event.

The discriminative validity comparing the three PEF groups with the EQ-5D-5L, AQL-5D and TTO showed no statistical significant differences across the groups, even though the mean rank scores generally increased as PEF severity reduced.

A sensitivity analysis using complete cases (participants completing all measures, at all time points) was also undertaken (see Supplementary Material Tables 1 to 6), where the results can be seen to be broadly comparable to that of the available case analysis.

### Comparison with other studies

We found the correlation coefficients between the TTO and the EQ-5D-5L were much weaker compared to the EQ-5D-5L and the AQL-5D. Even as the study progressed, they stayed weak with no statistical significance. A previous asthma-related cross-sectional study compared the TTO with the EQ-5D but had a higher correlation coefficient of 0.40, indicating a moderate correlation [33]. However, there were several differences between that study [33] and this current study around the participant population group, the questioning of the TTO and the number of levels on the EQ-5D. Nevertheless, in both studies, the TTO correlations had lower correlations compared to the other preference-based measures, which suggests the TTO is less suitable in asthma populations.

The discriminative test indicated that the preference-based measures were not very good at discriminating between the three PEF groups. Possible explanations for this result are that the preference-based measures are not able to capture changes related to the PEF, or that such PEF changes do not constitute a notable score change on the utility scale.

When assessing the responsiveness test conducted in this study (using the recovery questions from the participants at week 4 of the study), the AQL-5D and the EQ-5D-5L had larger responsiveness compared to the TTO. As the AQL-5D is derived from the AQLQ and has been also shown to have strong correlations in this study and others [21], this supports the use of the AQL-5D.

### Strengths and limitations

The use of three different PROMs in this study is a strength for the comparison of psychometric techniques. Despite this, only construct validity and responsiveness were assessed in this study, which is a limitation, as there are other psychometrics techniques which are useful for comparing measures. Secondly, the sample size was small when testing for discriminant validity and responsiveness, particularly due to the response rates. Therefore, it is recognized that a larger sample size would encourage more robust and representative results between groups.

### Recommendation for the future

In the light of the findings from this study, both the construct validity and responsiveness tests suggest which preference-based measures should be considered for the acute asthma population group for the criteria assessed. Overall, given the TTO performed poorly on the validity and responsiveness tests, we consider the AQL-5D and the EQ-5D-5L for use in economic evaluations for asthma studies. Even though the AQL-5D is a recent development [34], previous literature and this current study have strongly confirmed its performance [21, 35]. Therefore, it is suggested that the disease-specific questionnaire, AQLQ, is used in asthma studies in order to estimate utilities using the AQL-5D. However, given that NICE has recommended using the EQ-5D in economic evaluations [36, 37], this should still be considered.

## Conclusion

In conclusion, this study aimed to compare the performance between three preference-based measures that were used in a prospective cohort study, which estimated the loss associated with an asthma-related crisis event. Psychometric techniques, in particular, convergent validity, discriminative validity and responsiveness, were used in this analysis. In this study, out of the three outcome measures tested, we do not consider the TTO to be the most appropriate measure in this population given it performs poorly on tests of construct validity and responsiveness. Instead, we suggest the AQL-5D and EQ-5D-5L should be considered.

The EQ-5D-5L and the AQL-5D illustrated moderate to strong correlations throughout all three time points at baseline, week 4 and week 8. Small to large changes were observed in the preference-based measures for the level of sensitivity to change for the recovery groups. However, the discriminative test indicated that the preference-based measures were not very good at discriminating against the three PEF groups, and the TTO showed weak correlations between the EQ-5D-5L and the AQL-5D.

Therefore, the results overall highlight that the EQ-5D-5L and AQL-5D are well correlated and sensitive to change for participants who have had an asthma-related crisis event. For the purposes of economic evaluation studies, and the fact that previous research recommends the use of the EQ-5D, both the EQ-5D-5L and the AQL-5D should be considered in the future. Nevertheless, it is important to bear in mind that both of these questionnaires have been recently developed, and therefore, further research is encouraged on a larger, more complete data set.

## Electronic supplementary material

Below is the link to the electronic supplementary material.

Supplementary material 1 (DOCX 44 kb)
