# Supplementary material for: The construct validity and responsiveness of the EQ-5D-5L, AQL-5D and a bespoke TTO in acute asthmatics
Source: Qual Life Res. 2019 Nov 2;29(3):619–27. doi: 10.1007/s11136-019-02345-z (PMC7028833; doi:10.1007/s11136-019-02345-z)
Supplement: Supplementary file 1 — Supplementary material 1 (DOCX 44 kb) [file 11136_2019_2345_MOESM1_ESM.docx]

# **The construct validity and responsiveness of the EQ-5D-5L, AQL-5D and a bespoke TTO in acute asthmatics**

**Journal name: Quality of Life Research**

**Supplementary material**

**Authors:**

Christina-Jane Crossman-Barnes (MSc)^1^, Tracey Sach (PhD)^1^, Andrew Wilson (PhD)^1^, Garry Barton (PhD)^1^

**Author Affiliations:**

^1^Norwich Medical School, University of East Anglia, UK

**Correspondence:**

Christina-Jane Crossman-Barnes, University of East Anglia, Faculty of Medicine and Health Sciences, Norwich Medical School, Norwich, NR4 7TJ, UK

[C.Crossman-Barnes@uea.ac.uk](mailto:C.Crossman-Barnes@uea.ac.uk)

**Table 1: Baseline characteristics of participants for complete case analysis**

| Demographics | N = 44 |
| --- | --- |
| Age (mean, years) | 52.98 |
| Height (mean, cm) | 168.00 |
| Weight (mean, kg) | 91.62 |
|  |  |
| Gender (%) |  |
| Male | 25.00 |
| Female | 75.00 |
|  |  |
| Ethnicity (%) |  |
| White | 95.45 |
| White Other | 4.55 |
|  |  |
| Smoking Status (%) |  |
| Never | 40.91 |
| Smoker | 6.82 |
| Ex-Smoker | 52.27 |
|  |  |
| Highest Level of Education (%) |  |
| School | 40.91 |
| College | 38.64 |
| Degree | 20.45 |
|  |  |
| Employment status (%) |  |
| Full-time | 31.82 |
| Part-time | 9.09 |
| Retired | 29.55 |
| Stay at home parents | 6.82 |
| Student | 4.55 |
| Unemployed | 18.18 |

**Table 2: Convergent validity at baseline using Spearman’s rank Correlation coefficient**

|  | EQ-5D-5L (utility) | AQL-5D (utility) | TTO (utility) |
| --- | --- | --- | --- |
| EQ-5D-5L (utility) | N = 44  1.0000^a^ |  |  |
| AQL-5D (utility) | N = 44  **0.4003^a^**** | N = 44  1.0000^a^ |  |
| TTO (utility) | N = 44  0.1378^a^ | N = 44  0.2178^a^ | N =44  1.000^a^ |

^a^Pairwise correlation coefficients displayed.

^a^Correlation coefficients considered < 0.3 are weak, 0.3 to 0.5 are moderate and >0.5 are strong.

**p-value is < 0.01 therefore statistically significant at the 1% level.

**Table 3: Convergent validity at week 4 using Spearman’s rank Correlation coefficient**

|  | EQ-5D-5L (utility) | AQL-5D (utility) | TTO (utility) |
| --- | --- | --- | --- |
| EQ-5D-5L (utility) | N = 44  1.0000^a^ |  |  |
| AQL-5D (utility) | N =44  **0.4726^a^**** | N = 44  1.0000^a^ |  |
| TTO (utility) | N = 44  0.1805^a^ | N = 44  **0.3131^a^*** | N = 44  1.000^a^ |

^a^Pairwise correlation coefficients displayed.

^a^Correlation coefficients considered < 0.3 are weak, 0.3 to 0.5 are moderate and >0.5 are strong.

*p-value is < 0.05, **p-value is < 0.01 therefore statistically significant at the 5% level and 1% level respectively.

**Table 4: Convergent validity at week 8 using Spearman’s rank Correlation coefficient**

|  | EQ-5D-5L (utility) | AQL-5D (utility) | TTO (utility) |
| --- | --- | --- | --- |
| EQ-5D-5L (utility) | N = 44  1.0000^a^ |  |  |
| AQL-5D (utility) | N =44  **0.6118^a^**** | N = 44  1.0000^a^ |  |
| TTO (utility) | N = 44  0.0775^a^ | N = 44  **0.3114^a^*** | N =44  1.000^a^ |

^a^Pairwise correlation coefficients displayed.

^a^Correlation coefficients considered < 0.3 are weak, 0.3 to 0.5 are moderate and >0.5 are strong.

*p-value is < 0.05, **p-value is < 0.01 therefore statistically significant at the 5% level and 1% level respectively.

**Table 5: Discriminative (Known-groups) Validity at baseline using three PEF subgroups^a^ against preference based measures**

|  | < 50% of best / predicted PEF  Mean  Rank^b^ | < 50% of best/predicted PEF  N | 50-75% of best/predicted PEF  Mean  Rank^b^ | 50-75% of best/predicted PEF  N | >75% of best/predicted PEF  Mean  Rank^b^ | >75% of best/predicted PEF  N | P-value* |
| --- | --- | --- | --- | --- | --- | --- | --- |
| EQ-5D-5L utility | 28.40 | 10 | 18.91 | 23 | 22.56 | 11 | 0.182 |
| AQL-5D utility | 30.25 | 10 | 19.15 | 23 | 21.00 | 11 | 0.078 |
| TTO utility | 16.50 | 10 | 19.09 | 23 | 20.44 | 11 | 0.546 |

^a^Kruskal-Wallis test conducted and PEF split into three subgroups: <50% of PEF best / predicted = life threatening / acute severe asthma; 50-75% of PEF best/predicted = moderate acute asthma and >75% of best/predicted asthma for good/very good asthma (1).

^b^Mean rank: The utilities are ranked from lowest to highest, and an average is taken based on the ranked order of utilities. The group with the lowest mean rank have the greatest number of lower utility values, and the group with the highest mean rank have the greatest number of higher utility values.

*No statistical significant difference found for all PROMs between the three PEF subgroups.

**Table 6: Responsiveness of all preference based measures between baseline and week 4 for recovery rates**

| Items | N | Baseline (mean) | Week 4 (mean) | Mean change | SD at baseline | SD at change | ES^b^ | SRM^c^ | P value^a^ |
| --- | --- | --- | --- | --- | --- | --- | --- | --- | --- |
| EQ-5D-5L |  |  |  |  |  |  |  |  |  |
| Very good | 10 | 0.820 | 0.907 | 0.087 | 0.219 | 0.126 | 0.397 | 0.690 ** | **0.028** |
| Good | 12 | 0.598 | 0.818 | 0.221 | 0.337 | 0.270 | 0.656 | 0.819*** | **0.004** |
| Moderate | 15 | 0.607 | 0.722 | 0.115 | 0.274 | 0.213 | 0.420 | 0.540* | 0.083 |
| Poor | 7 | 0.641 | 0.313 | -0.328 | 0.114 | 0.242 | -2.877 | -1.355 *** | **0.018** |
| AQL-5D |  |  |  |  |  |  |  |  |  |
| Very good | 10 | 0.544 | 0.537 | 0.007 | 0.117 | 0.021 | 0.060 | 0.333 * | **0.011** |
| Good | 12 | 0.612 | 0.790 | 0.178 | 0.144 | 0.141 | 1.236 | 1.262 *** | **0.005** |
| Moderate | 15 | 0.552 | 0.612 | 0.060 | 0.091 | 0.131 | 0.659 | 0.458 * | 0.147 |
| Poor | 7 | 0.544 | 0.537 | -0.007 | 0.117 | 0.021 | -0.060 | -0.333* | 0.373 |
| TTO |  |  |  |  |  |  |  |  |  |
| Very good | 10 | 0.679 | 0.932 | 0.254 | 0.250 | 0.329 | 1.016 | 0.772 ** | 0.074 |
| Good | 12 | 0.650 | 0.905 | 0.255 | 0.269 | 0.376 | 0.948 | 0.678 ** | 0.261 |
| Moderate | 15 | 0.608 | 0.707 | 0.098 | 0.299 | 0.377 | 0.328 | 0.260 * | 0.095 |
| Poor | 7 | 0.864 | 0.795 | -0.069 | 0.184 | 0.254 | -0.375 | -0.272* | 0.859 |

^a^Wilcoxon signed-rank test conducted and p-values in bold are statistically significant at the 5% level.

^b^ES = Effect size (mean change / SD at baseline)

^c^SRM = Standardized response mean (Mean change / SD of change). If SRM = 0.2 to 0.50 equals small, 0.50 to 0.80 equals moderate and 0.80 and above equals large.

*small change, small responsiveness

**moderate change, moderately responsive

***large change, largely responsive

**References**

1. British Thoracic Society. Scottish Intercollegiate Guidelines Network. British guideline on the management of asthma: A national clinical guideline 2016.
